# Rapidly fatal outcome of Covid-19 after successful emergency surgery during pandemic outbreak in Northern Italy

**DOI:** 10.1016/j.ijscr.2020.06.073

**Published:** 2020-06-20

**Authors:** Filippo Montali, Gerardo Palmieri, Lorenzo Casali, Lorenzo Pagliai, Renato Costi

**Affiliations:** a Unità Operativa di Chirurgia Generale, Ospedale di Vaio, Fidenza (Parma), Azienda Sanitaria Locale di Parma, Italy; b Dipartimento di Medicina e Chirurgia, Università di Parma, Parma, Italy

**Keywords:** Pandemic outbreak, COVID-19, SARS CoV-2, Emergency surgery, Fatal outcome

## Abstract

**Introduction:**

A pandemic outbreak of novel coronavirus, named SARS-CoV-2 and responsible of Coronavirus Disease 2019 (COVID-19), has rapidly spread from China to Europe, being Northern Italy the first focus outside Asia. Little is known about the evolution of SARS-CoV-2 infection in patients undergoing surgery.

**Presentation of case:**

Here we report the first confirmed case of early postoperative SARS-CoV-2 infection in a patient recovering after Hartmann’s procedure for acute diverticulitis. After an otherwise unevenful postoperative course, on post-operative day 8, the patient suddenly presented hyperpyrexia and cough, rapidly evolving to respiratory failure and death 36 h after symptoms onset. CT-scan identified bilateral, diffuse, interstitial pneumonitis and oropharyngeal swab test confirmed the presence of SARS-CoV-2. A previous contact with the partner, developing the same symptoms, remained unrecognized until ICU admission.

**Discussion:**

During a pandemic outbreak, the early identification of SARS-CoV-2 infection of an inside patient initially considered to be infection-free has a pivotal importance not only for the prompt patient’s management, but also to avoid infection spreading to other patients and hospital personnel.In the reported case, a more precise information to the patient regarding the imperative necessity to inform the medical personnel of any person of his entourage presenting, at any time, any tell-tale sign, symptom or examination which may be attributed to COVID-19, may have had allowed to anticipate patient’s isolation and examinations and procedures aimed at identifying such an infection.

**Conclusion:**

Persistent hyperleucocytosis and increased CRP serum level in spite of uneventful postoperative course were the only, aspecific markers of an ongoing SARS-CoV-2 infection before symptoms’ onset, and should be considered in future clinical practice in order to adopt the prompt and appropriate patient management and limit COVID-19 contagion in surgical units.

## Highlights

• The first focus outside Asia of Coronavirus disease is Northern Italy.
• Patients undergoing emergency surgery can develop SARS-CoV-2 more easily.
• The outcome of these patients can be very poor despite a uneventful POD.
• Early diagnosis of Covid-19 infection is essential to improve the prognosis and reduce the risk of contagion among hospital staff.

## 1 Introduction

A pandemic outbreak of interstitial pneumonia caused by novel coronavirus, named SARS-CoV-2 and responsible of Coronavirus Disease 2019 (COVID-19), was first notified in Wuhan, China, and has spread rapidly in China, Corea, Japan and Europe, being Northern Italy the first pandemic focus outside Asia [1]. COVID-19 infection has been reported to have a 3.9% mortality in Chinese population, whereas in Italy fatalities has reached 9.2% (updated on March 26, 2020) [2] Targeting mostly elderly patients with comorbidities, COVID-19 presents a typical clinical pattern including aspecific onset with flu-type symptoms (cough and hyperpirexia, less frequently anosmia, ageusia, congiuntivitis). Such a clinical picture may evolve to progressive respiratory function deterioration, at times needing ICU management and mechanichal ventilation, and, in a non-negligible number of patients, leading to death by respiratory failure. To date, old age and comorbidities have been suggested as predictors of poor prognosis of patient developing COVID-19 [3].

Colonic diverticulosis (the presence of colonic diverticula, usually in the sigmoid colon) is a common, multifactorial condition in Western countries, affecting half the population over the age of 60 [4]. Acute diverticulitis (AC) is the most frequent complication of colonic diverticulosis, and a common cause of emergency hospitalization/operation in the elderly population [5]. Here we report the first confirmed case of SARS-CoV-2 early postoperative infection occurring in a patient recovering after Hartmann’s procedure for perforated sigmoid diverticulitis. Since little is known about the evolution of SARS-CoV-2 infection in patients undergoing surgery, the analysis of the present case with its rapid evolution through disease’s stages and death, and the intrinsic implications regarding the contagion to hospital personnel, may have implications in future clinical practice. This case report has been drafted according to recent guidelines [6].

## 2 Presentation of case

**Fig. 1:**
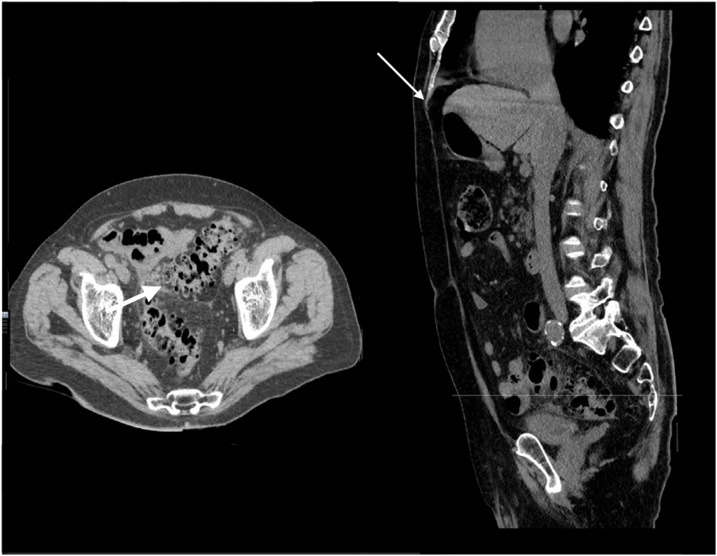
Preoperative abdominal CT scan showing complicated acute diverticulitis of the sigmoid colon (thick arrow) and free intraperitoneal air (thin arrow).

An 83-year-old man presented to our department with one-day history of increasingly severe, continuous lower abdominal pain, without hyperpirexia, nausea or vomit. At this time, an increasing but still limited number of newly diagnosed COVID-19 patients were admitted on another floor of the same hospital. Patient’s comorbidities included chronic obstructive pulmonary disease (COPD), arterial hypertension chronic and ischemic heart disease, previously treated by coronary angioplasty. At physical examination, the patient was eupnoic, blood pressure was 140/85 mmHg and heart rate 100 beats/minute, the abdomen was bloated and presented diffuse rebound tenderness. Blood tests showed hyperleucocytosis (WBC count: 20 × 10^3^/μL) with neutrophilia (90%), and increased CRP (264 mg/L), without any other relevant alteration. Abdominal CT-scan showed free air, liquid collections, and severe inflammatory thickening of the sigmoid colon, presenting multiple diverticula (Fig. 1).

Laparotomy confirmed acute sigmoiditis associated with diffuse purulent peritonitis, and Hartmann’s procedure (sigmoid resection and descending colon terminal stoma) was carried out. An otherwise uneventful postoperative course (first flatus on postoperative day (POD) 3, solid food resumption on POD 4, was marked by a persistent hyperleukocytosis, as WBC count ranged between 17 × 10^3^/μL and 20 × 10^3^/μL through POD 8, despite antibiotic therapy (piperacillin/tazobactam and metronidazole).

**Fig. 2:**
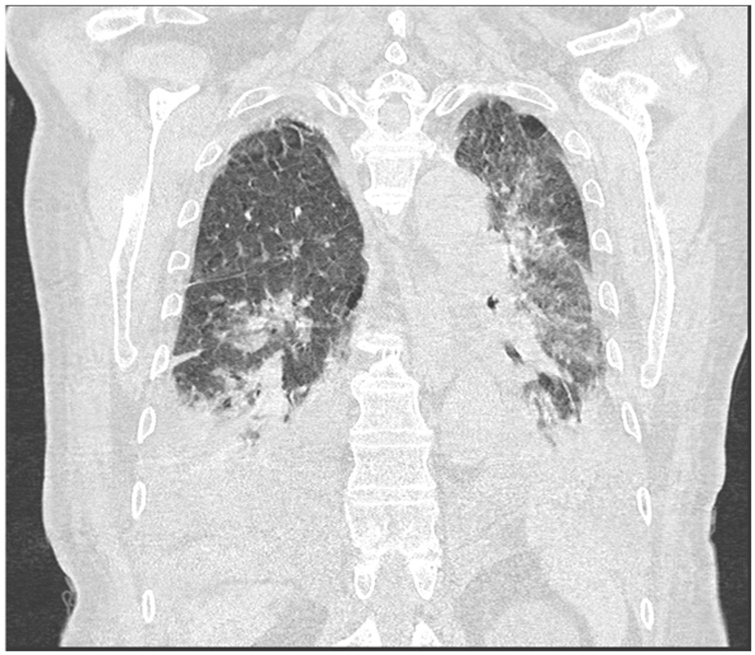
High-resolution computed tomography scan of lung showing extensive bilateral interstitial pneumonitis.

On POD 8 afternoon, the patient suddenly presented dry cough, sore throat, dyspnea and hyperpirexia (temperature: 38.5 °C) and hypoxemia (SaO_2_: 88–90%) under ambient air. As he was transferred to the ICU for ongoing acute respiratory failure, further imaging examinations were planned for the differential diagnosis, being lobar pneumonitis and/or COPD exacerbation the most likely etiologies suspected. Later that day, the patient reported that his 70-year-old partner, one week before (on patient’s POD 2), presented cough and mild hyperpyrexia (T: 37.8 °C), was admitted to another hospital and underwent oropharyngeal swab, which later (on POD 7) resulted positive for SARS-CoV-2. Our patient’s oropharyngeal swab, by real-time reverse-transcriptase-polymerase-chain-reaction (rRT-PCR), was then collected in accordance with WHO guidelines for the management of the current COVID-19 outbreak [7]. A high-resolution computed tomography (HRCT) scan identified extensive bilateral unusual interstitial pneumonitis (Fig. 2). Owing to rapidly deteriorating respiratory function, on POD 9, the patient underwent intubation and mechanical ventilation, initially in supine position and later in prone position. On POD 10, oropharyngeal swab test resulted positive for SARS-CoV-2. Few hours later, the patient died by respiratory failure refractory to any treatment.

Shortly after SARS-CoV-2 infection was suspected, on POD 9, a retrospective investigation of patient’s person-to-person contacts with medical and non-medical personnel was undertaken, in order to identify any possible further contagion. Three days after patient’s death, patient’s partner died by COVID-19-related respiratory failure, too. One week later, the oropharyngeal swab test of two members of our team resulted positive and three negative; the results of five more are expected.

## 3 Discussion

COVID-19 outbreak in 2020 spring is dramatically spreading worldwide [8]. The first person-to-person transmission in Northern Italy was confirmed on February 21, 2020, rapidly spreading into the largest COVID-19 outbreak outside Asia to date, as witnessed by over 73,380 infected people and 6801 fatalities in Italy by March 26, 2020, five weeks later. Old age and comorbidities have been suggested as predictors of poor prognosis of patient affected by COVID-19 [3,9], but little is known about patients undergoing surgery and postoperatively developing COVID-19, including how to suspect (and potentially screen) SARS-CoV-2 infection in a patient recovering from surgery. In fact, SARS-CoV-2 infection is typically asymptomatic until the onset of aspecific respiratory symptoms, which, by the way, may be attributed to several other causes after aggressive emergency colon surgery, and which consequently may be unrecognized or underestimated.

By the way, the management of AD depends on disease severity, which is assessed by Hinchey classification [5]. While uncomplicated episodes (Hinchey stage I) are managed conservatively, the recommended management of acute diverticulitis associated with diffuse purulent or stercoral acute peritonitis (Hinchey III and IV), is surgery [5]. Hartmann’s operation is indicated for the most severe cases, which are associated with non-negligible morbidity/mortality [10,11].

The reported patient, an 83-year-old gentleman with comorbidities, represents the typical target of SARS-CoV-2. Severe AC and aggressive surgery may be thought to have played a role in such a dramatic evolution of COVID-19 interstitial pneumonitis from asymptomatic infection, to symptoms’ onset to respiratory failure, since surgery-related immunosuppresion is diffusely considered a significant risk factor for developing respiratory tract infections [12,13,10]. On the other hand, interestingly, SARS-CoV-2 infection seemingly did not affect surgery outcome in this particular case, as the patient had his first flatus on POD 3, resumed solid food on POD 4, and postoperative course was uneventful until respiratory breakdown.

It is debatable whether an earlier diagnosis may have allowed avoiding such a rapid clinical degradation and death, nevertheless, some considerations seem worthwhile. During a pandemic outbreak, the early identification of SARS-CoV-2 infection of an inside patient initially considered to be infection-free has a pivotal importance not only for the prompt patient’s management, but also to avoid infection spreading to other patients and hospital personnel. In the reported case, a more precise information to the patient regarding the imperative necessity to inform the medical personnel of any person of his entourage presenting, at any time, any tell-tale sign, symptom or examination which may be attributed to COVID-19, may have had allowed to anticipate patient’s isolation and examinations and procedures aimed at identifying such an infection. Secondarily, the persistent increase of aspecific inflammatory markers (hyperleucocytosis and increased CRP), which were not justified by the uneventful postoperative course, were for days the only warning sign before COVID-19 symptoms’ onset. Although the typical laboratory finding of viral infection is lymphopenia in the non-operated patient [14], little is known about COVID-19 after surgery. We believe that, with an ongoing pandemia and considering patient's old age and comorbidities, those findings should have prompted to anticipate imaging examination, oropharyngeal swab and patient isolation, even without typical symptoms, although such a policy is at present not recommended for asymptomatic outside patients or personnel [8]. Since the first reports from China [[7], [8], [9]], high-resolution pulmonary CT-scan is reported to show the patognonomic aspect of interstitial bilateral pneumonia, thus rapidly becoming the mainstay of COVID-19 early diagnosis while waiting for SARS-CoV-2 confirmation by oropharyngeal swab.

## 4 Conclusion

The reported one is the first confirmed case of COVID-19 interstitial pneumonitis early after a surgical procedure, dramatically leading to death in a matter of hours and possibly causing viral infection further spread in hospital personnel. The collection of a simple information regarding a member of patient's entourage developing COVID-19, and the unjustified persistence of hyperleucocytosis and increased CRP serum level (in spite of uneventful postoperative course), may have allowed anticipating by days the diagnosis of an ongoing SARS-CoV-2 infection. Our observations should be considered in future protocols during COVID-19 outbreak defining clinical practice aimed to identify affected patients and to limit viral spread in surgical units.

In conclusion, during a pandemic outbreak such as that caused by COVID-19, surgeons must systematically question any patient about possible contagious contacts and rule out viral infection as a possible cause of unjustified changes from otherwise uneventful postoperative course after apparently successful surgery.

## Declaration of Competing Interest

None.

## Sources of funding

None reported.

## Ethical approval

Ethical approved as a Case Report.

## Consent

Written informed consent was obtained from the patient's relatives for publication of this case report and accompanying images. A copy of the written consent is available for review by the Editor-in-Chief of this journal on request.

This work respects the complete privacy of the patient involved and maintains absolute anonymity.

## Author contribution

Filippo Montali : writer of the paper and study concept.

Gerardo Palmieri and Lorenzo Pagliai : data collector and references.

Renato Costi e Lorenzo Casali : design of the paper and figures.

## Registration of research studies

https://www.researchregistry.com/browse-the-registry#home/registrationdetails/5eb4018126414100151e2b44/.

researchregistry 5578.

## Guarantor

Filippo Montali MD, University of l’Aquila, Italy.

Renato Costi MD, PhD, FACS, University of Parma, Italy.

## Provenance and peer review

Not commissioned, externally peer-reviewed.
